# Sleep Matters: Unpacking the Link between Sleep Disorders and Clinical Characteristics in Schizophrenia.

**DOI:** 10.1192/j.eurpsy.2024.1530

**Published:** 2024-08-27

**Authors:** K. Razki, A. Larnaout, C. Najar, S. Ben Aissa, R. Lansari, W. Melki

**Affiliations:** Psychiatry department, razi hospital, manouba, Tunisia

## Abstract

**Introduction:**

Sleep disorders are a significant concern for patients with schizophrenia, and they can have a profound impact on their quality of life. Studies have shown that sleep disturbances are prevalent in patients with schizophrenia, and they may be linked to the clinical characteristics of the disorder. Despite this, the exact nature of the relationship between sleep disorders and schizophrenia remains unclear. Understanding this relationship is critical as it may lead to better diagnosis and treatment of both conditions, ultimately improving the overall health and wellbeing of patients.

**Objectives:**

To establish the link between sleep disorders and clinical characteristics in a clinical population being treated for schizophrenia.

**Methods:**

We conducted a cross-sectional, descriptive, and analytical study that took place over a period of one month (from 1st to 31nd March 2023) among patients consulting the post-care service of Psychiatry Department D at Razi Hospital, Tunisia. We included patients aged between 18 and 65 years, diagnosed with schizophrenia according to DSM-5, and stabilized on psychiatric treatment. We used the Pittsburgh Sleep Quality Index (PSQI) to evaluate sleep quality over a period of one month. The evaluation of the clinical characteristics of schizophrenia was carried out using the Positive and Negative Syndrome Scale (PANSS).

The interview was conducted by a single researcher, and when the questionnaire was distributed to the participants, we explained the framework and the principle of this study as well as the implications of participating in it and explained that the participant could stop participating at any time if he or she wished.

**Results:**

We collected data from 30 male patients with a mean age of 42.5 ± 14.02. The mean overall PSQI score was 9.23 ± 4.58. The subscales evaluating the subjective quality of sleep obtained an average score of 1.42 ± 0.72, sleep latency was 1.61 ± 1.33, sleep duration was 1.01 ± 0.98, habitual sleep efficiency was 0.67 ± 0.75, sleep disturbances were 0.91 ± 0.52, sleep medication use was 1.36 ± 1.68, and daytime dysfunction was 1.12 ± 0.96. The mean scores of PANSS were: positive scale (28.26 ± 5.93), negative scale (18 ± 6.15), and general psychopathology scale (90.03 ± 16.21). We found a statistically significant association between the positive PANSS scale and sleep latency (p=0.002) and sleep medication use (p<10-3).

**Conclusions:**

The findings highlight the importance of evaluating and addressing sleep disturbances in the overall management of patients with schizophrenia, as they may have an impact on the severity of clinical symptoms.

**Disclosure of Interest:**

None Declared

